# Talent Identification and Development in Paralympic Contexts: Current Challenges

**DOI:** 10.3389/fspor.2022.926974

**Published:** 2022-06-17

**Authors:** Nima Dehghansai, Ross A. Pinder, Joe Baker

**Affiliations:** ^1^Paralympic Innovation, Paralympics Australia, Adelaide, SA, Australia; ^2^Kinesiology and Health Science, York University, Toronto, ON, Canada

**Keywords:** para sport, athletes with impairment, pathway, selection, expertise

## Abstract

This short review explores the state of talent identification and development of athletes in Paralympic contexts. While talent identification typically occurs during adolescence, this practice is more complex and variable in Paralympic contexts compared to non-Paralympic contexts. For example, Paralympic athletes can have impairments that are congenital or acquired at any time across their lives. Therefore, they can enter performance pathways at unpredictable times. Furthermore, differences and nuances associated with athlete impairments (type and severity), compounded by other systematic complexities (e.g., systems of classification) highlight the need to consider alternative and creative approaches to talent identification and development. We provide an overview of some of these complexities, highlight areas for future research, and provide recommendations for practitioners.

## Introduction

Talent identification and development has been forefront of discussions in the practical and scientific realm for quite some time. At the core of these discussions remains a lack of clarity around a universal definition of “talent” (Dohme et al., 2017; Baker et al., 2019). Many scientists argue that without a clear understanding of what talent *is*, it becomes difficult to identify and develop *it*, but more importantly, to track it and evaluate our efficiency and effectiveness of the factors identified as *talent* (Issurin, 2017; Baker et al., 2018). It appears that we are not particularly good at identifying “talent” (Till and Baker, 2020), or predicting future potential based on current indicators (Güllich, 2014; Baker et al., 2019). In Paralympic contexts, our scientific base and understanding is further challenged, with much of the existing knowledge being in informed by work that is completed outside these contexts. While it may be argued that organizations in Paralympic contexts face some of the same challenges as those in other contexts (e.g., lack of clarity on operational definition of talent, challenges in forecasting future performance on current indicators), there are specific constraints that introduce additional challenges. Primarily, the underpinning challenges reside in relation with impairment-related factors which can have a marked impact across micro- (i.e., athlete-specific or directly related to, including classification, program and facility availability and accessibility) and macro-levels (broader societal landscape pertaining to policy, infrastructure, funding, and resource allocation, Radtke and Doll-Tepper, 2014; Patatas et al., 2018; Dehghansai et al., 2020). The aim of this short review is to introduce two primary factors that are pertinent to talent identification and development, that is, impairment onset and classification (and by extension type of impairment). In the process we will also highlight two systemic constraints: limited pools of athletes and funding, which influence the dynamics of the athlete development environment. Finally, we provide recommendations for researchers and practitioners, including resources for coaches and talent identifiers.

## Impairment-Related Factors

### Impairment Onset

Research has identified that athletes with congenital impairments have marked differences in developmental trajectories compared with athletes with acquired impairments (Dehghansai et al., 2017; Patatas et al., 2018). Athletes in Paralympic contexts enter sport systems at various stages in their careers due to the varied onset of impairment (Radtke and Doll-Tepper, 2014) andimpairment onset represents a key marker to reference across athletes' careers. This is an important frame of reference to understanding how trajectories are shaped. For example, an athlete with an impairment acquired in adulthood may have extensive experience in non-Paralympic sports, prior to pursuing a high-performance career in Paralympic contexts. This may differ from a younger athlete with a congenital impairment interested in recreational sport, and/or an athlete with congenital impairment looking to embark on a journey to becoming an elite athlete with little to no previous sporting experience.

In extension of this work, Dehghansai et al. (2021b) examined the variation in athletes' careers based on when they acquired their impairment. The authors categorized athletes into groups representing different biological maturation phases to better understand the interaction between athletes' impairment and the phase during development that they acquire their impairment. Findings highlighted high degrees of variation in athletes' sporting careers based on the onset of their impairment. More specifically, athletes with congenital or early acquired impairments reached milestones at a younger age; however, athletes with later acquired impairments (i.e., early adulthood or adulthood) progressed through these milestones at a faster pace. Groups also had different training profiles, with changes to how much time was invested in different training types (sport-specific, physical preparation, mental preparation, etc.) and settings (with a coach and other teammates or alone, etc.). Furthermore, athletes with experience in other sports (both non-Paralympic and Paralympic) reported participating in sports that were similar to their current sport.

Previous research findings, including the impact of impairment-onset, are crucial to understanding and improving the quality of the developmental environment across the pathway. First, it highlights the array of issues that need to be considered for athlete recruitment, identification and/or transfer (Dehghansai and Baker, 2020; Patatas et al., 2020). More specifically, where athletes are in their sporting career (i.e., their sport/training age) will differ from their chronological age, and, therefore, the experiences they bring to the sport will vary. Relatedly, the type of sport they had experience in could affect their abilities and their “baseline” in their new sport (Dehghansai et al., 2021b). Second, once an athlete enters a performance pathway, the type of resources necessary to support optimal development can vary based on their readiness (Dehghansai et al., 2020, 2021b). This includes impairment-specific considerations (e.g., equipment, accessibility of venues, etc.) as well as the type/style of coaching they require (Bentzen et al., 2020; Dehghansai et al., 2021c), their preference for a type of training profile (Dehghansai et al., 2021b), and so forth. These elements should be taken into consideration when developing policies and guidelines for resource allocation and athlete support. A challenge many stakeholders in the Paralympic context face, given the limited funding and accessibility to resources (Dehghansai et al., 2021d; Patatas et al., 2021, 2022).

### Classification

With the aim of “keeping a level playing field” (e.g., similar to how many sports may use age, weight, and sex categorizations), Paralympic sport utilizes classification systems to better organize athletes with similar levels of activity limitation as a result of their physical, vision, or intellectual impairments. While this is exclusionary (there are only a set number of eligible impairments and classifications within a sport), at the competitive level, it is necessary to provide (or at least attempt) a competition environment that is fair and evidence based. Like impairment onset, data have suggested variations in athlete impairment type influences performance trajectories and training histories (Dehghansai et al., 2021a). Similarly, coaches and high-performance personnel have highlighted how athletes' impairment type and severity, and, therefore, their *potential* classification is used as a key indicator for initial identification and successful development (Dehghansai et al., 2021c; Patatas et al., 2022). Indeed, it has been highlighted that one of the key skills (and challenges) for Paralympic coaches and other support personnel is the ability to be able to anticipate which class an athlete will potentially be classified in Radtke and Doll-Tepper (2014), Mann et al. (2017), and Dehghansai et al. (2021c). While provisional classification (i.e., a quick prediction of an athlete's classification) can and does occur in many domestic contexts, athletes are required to be classified officially, at an international event. There are clearly risks associated with this; for example, an athlete may spend extensive time (e.g., training hours) developing in a sport, with that sport investing significant resources, only for the athlete to eventually be found to be ineligible for a given Paralympic sport class either due to inaccurate initial provisional classification, changes in the athlete's impairment, or a change in the criteria used to determine classification.

Scenarios may also arise where an athlete is classified in what is perceived as an “unfavorable” class (i.e., they are at the “lower” end of their class when considering the severity of their impairment in comparison to other athletes in that class). Athletes classified in the higher end of a class may be given more resources (more coaching, access to camps, etc.) which further supports their development in a cyclical relationship where effects are magnified over time (similar to evidence found with relative age effect highlighting the consequential benefits for athletes with earlier maturation onset, for a review, see Wattie et al., 2015). In addition, certain sports may aim to be strategic and identify classes that are less competitive internationally, or target athletes closer to class “cut offs” which introduces an additional layer of complexity regarding the most appropriate athlete for a given sport, at a given time. Thus, classification system and by extension, athletes' impairment add to the complexity of forecasting athletes' future performance in sport.

## Other Factors

### Resources

Paralympic sports have historically had less funding compared to their non-Paralympic counterparts which extends to limited resources supporting Paralympic sport athletes' development (Martin-Ginis et al., 2016; Patatas et al., 2020). While sports are already an expensive participation activity (e.g., travel and competition expenses, expenses associated with private coaching and access to training facilities, team registration fees, etc.), the additional costs associated with impairment-related factors exacerbates athletes' circumstances (e.g., equipment cost including prosthetics or wheelchairs, travel for classification). Furthermore, some athletes have higher support needs and are dependent on caregivers or parents' assistance for access to training facilities and travel domestically and abroad for camps or competitions. The challenges related to athlete wellbeing and care introduce an additional layer of obstacles for athletes' participation in sports (Goodridge et al., 2015). Because of the typically smaller pool of athletes in Paralympic sports compared with non-disabled sports, and the limited competitive opportunities domestically, exposure to international competition can be seen paramount to athletes' development. Impairment-related factors compound these costs (i.e., equipment cost, accessible infrastructure, accommodation and flight costs, classification, support needs, etc.), and the number of athletes a sport can support is inevitably reduced.

In addition, while the Paralympic Games have become a globally recognized event, this increased appreciation and recognition has not generally resulted in meaningful differences in incentives for Paralympic sports domestically. Therefore, sports must be strategic with how they use their funding in creating environments to maximize the potential for their athletes. At times, the limited resources result in less athletes being supported through sports, and the athletes that are unable to fund their own sporting journeys are left with little chance for exposure to high-performance training facilities, camps and/or competitions that are invaluable to their development. Limited funding also constrains sports from being able to best support coaches and their development. With resources scarce, sports are not able to monitor and expand on key sport-related components including data tracking and analysis, program development, or educational resources that could help athletes, coaches, and practitioners. Relatedly, there are challenges to maintaining an optimal group of support staff to surround the athlete and coach (e.g., physiotherapists, psychologists, etc.). Therefore, sports tend to find strategic ways to either support the coaches and other practitioners in their organizations or most often, are under-staffed and overworked with limited resources to support their developments (Patatas et al., 2018; Dehghansai et al., 2019).

### Athlete Pool

Given classification is an exclusionary process, selecting athletes who are (a) eligible for classification, and (b) good “bets” for future success has merits. The consequences of this approach are felt when considering the number of *potential* athletes for a specific sport since not all persons with a given impairment are interested in participating in sports generally, let alone at the high-performance level. The challenge of identifying potential athletes is exacerbated by the limited resources a sport has and the type of athlete they choose to support. While more mature sports with a history of established programs and a wider classification system (e.g., Para athletics or Para swimming) may have less difficulty recruiting athletes into the system, they too, will have to be strategic in which athletes they select based on issues related to athletes' potential given their classification, the pool of depth in that class, and so forth. Even within these sports, there are certain classifications that have limited numbers of athletes involved. The two athlete cohorts that are visibly less involved in Paralympic sports are athletes with high support needs and female athletes (Dehghansai and Baker, 2020; Lowry et al., 2022). While the reasons underpinning athletes with high support needs' lack of participation in sport is beyond the scope of this review (e.g., cost of participation, specialized equipment, travel cost, lack of inclusive and accessible environments, qualified coaches/staff, tailored programs; Goodridge et al., 2015), these barriers lead to having less coaches and athletes involved in these classes. Similarly, the inclusivity of the environment along with intersection of other social, personal, and cultural factors have been identified as reasons for lack of female athlete participants in the Paralympic contexts (Shakib, 2003; Dehghansai and Baker, 2020; Dehghansai et al., 2021c).

## Application for Practitioners

Given the complexity of talent identification and development, the ideal approach would be to delay the exclusionary process (i.e., sport classification) and instead allow athletes with different abilities to participate in sports as long as possible. However, given funding limitations (and consequently impacting staffing and resources), this approach is not feasible for many Paralympic sport organizations. In this section, we provide emerging ideas to extend the discussion of how practitioners currently approach talent identification and development in Paralympic settings. We recognize the importance of individual contexts, and that each environment will call for unique approaches and, thus, the purpose of this section is to stimulate “outside of the box thinking” rather than providing a concrete solution to the key challenges discussed above.

1. **Resource pooling:** There are many ways sports could pool resources, whether it is through collaboration with other sports, other stakeholders (e.g., including impairment-specific organization such as the International Blind Sport Federation), national organizations (e.g., National Paralympic Committees), or local and state networks (e.g., local clubs, state sport or impairment governing bodies) to identify strategies on how to utilize limited resources more effectively to address pervasive problems across the pathway. For example, strategies developed at the national level to support coaches working with high-performance athletes could be modified to meet the needs of coaches at earlier stages of the development pathway. Similarly, general framework recommendations developed by a Blind Sport Federation on how to work with athletes with visual impairment could be shared with all sports that have athletes with visual impairments. A shared resource model could also provide multi-sport access opportunities for athletes at earlier stages of their careers (e.g., multisport hubs). This would allow athletes opportunity to sample sports, while giving each sport a larger pool of athletes at the participation level. Furthermore, this could provide opportunities for cross-cultural development for coaches, and allow sports to delay or extend the selection process while providing development environments for athletes. Creating hubs of this nature could also incorporate provisional classification where guidance can be provided to sports with athletes pending official classification. While resources are scarce, pooling support staff across multiple sports may also enable flexibility and at the same time, ensure athletes receive a higher level of support for their continued participation and development.
2. **Formalized entry points and a flexible pathway:** Formalizing entry points at various points across the pathway could allow sports more flexibility in how resources are allocated to support athletes. This structure allows for better organization of resources, task distribution among staff, and increases effective communication and accountability within the network. The formalization could include better understanding of the system, where resources are located across the country, including protocols on how to integrate an athlete into the system while considering their expertise and where they would “sit” within the pathway. For example, if a high-performance athlete with experience in a non-Paralympic sport acquired an impairment and was joining Para cycling from BMX, ensuring there are formalized processes embedded into the pathway to support the athlete's transition from entry to integration and subsequent development is paramount. Steps to formalize the process could include (a) dedicated personnel to oversee the proceeding, (b) a streamlined athlete testing process, (c) identification of local clubs with structured mechanisms to support the athlete, (d) established communication line between the governing body and clubs to organize and facilitate the transition, (e) clear benchmarks for coaches and the athlete to understand the evaluation process and potential growth opportunities, and (f) transparent guidelines on resource allocation and facilitation. This formalized entry point could also facilitate a more effective transfer system, where athletes interested in switching sports at the high-performance level are given a platform to request and broadly explore other sports without fear of repercussion from, or impact on, their current sport (Dehghansai et al., 2022). Collaboration and open communication become paramount to the success of any of the initiatives whether it is recruitment or transfer, given the number of moving parts and organizations involved in the process.
3. **Network collaborations:** Sports could also look to universities and research centers for collaborations to gather data and expand key components pertaining to their sport. As alluded to in the previous recommendation, the importance of benchmarking, understanding athlete profiles, and being able to track and monitor progress are vital to the system improvement. Utilizing an array of scientists and trainees to gather, collate, disseminate findings can bypass resource capacity challenges, while at the same time, providing valuable opportunity for professional development of junior scholars. Moreover, embedding research teams into the sport allows for evidence informed decision making, which in turn, can help in improving the allocation of resources and support to the athletes and coaches (Dehghansai et al., 2019).

## Conclusion

While Paralympic sport contexts carry similar challenges to that of their non-Paralympic counterparts, there are additional complexities that Paralympic sport organizations must navigate. Specifically, these organizations have to be creative in how they design their programs considering the limited resources. Sharing of resources between sports on strategies can reduce costs associated with certain operational components (e.g., sharing of camp spaces, resource development, and coaching frameworks). Formalization of the entry points and network collaborations could further increase the efficiency and maximize the resources available to sports. Continuing to innovate and challenge to think “outside the box” could not only lead to solutions to immediate constraints but spark new ways of operating and managing systems.

## Author Contributions

All authors contributed to the development of the ideas, content, and editing of the manuscript, with the lead author taking the responsibility to collate, formulate, and finalize the paper.

## Conflict of Interest

The authors declare that the research was conducted in the absence of any commercial or financial relationships that could be construed as a potential conflict of interest.

## Publisher's Note

All claims expressed in this article are solely those of the authors and do not necessarily represent those of their affiliated organizations, or those of the publisher, the editors and the reviewers. Any product that may be evaluated in this article, or claim that may be made by its manufacturer, is not guaranteed or endorsed by the publisher.
